# Discovery, structure, mechanisms, and evolution of protein-only RNase P enzymes

**DOI:** 10.1016/j.jbc.2024.105731

**Published:** 2024-02-08

**Authors:** Walter Rossmanith, Philippe Giegé, Roland K. Hartmann

**Affiliations:** 1 Center for Anatomy & Cell Biology, Medical University of Vienna, Vienna, Austria; 2 Institute for Plant Molecular Biology, IBMP-CNRS, University of Strasbourg, Strasbourg, France; 3 Institute of Pharmaceutical Chemistry, Philipps-University Marburg, Marburg, Germany

**Keywords:** RNase P, transfer RNA, protein-only RNase P, PRORP, HARP

## Abstract

The endoribonuclease RNase P is responsible for tRNA 5′ maturation in all domains of life. A unique feature of RNase P is the variety of enzyme architectures, ranging from dual- to multi-subunit ribonucleoprotein forms with catalytic RNA subunits to protein-only enzymes, the latter occurring as single- or multi-subunit forms or homo-oligomeric assemblies. The protein-only enzymes evolved twice: a eukaryal protein-only RNase P termed PRORP and a bacterial/archaeal variant termed homolog of *Aquifex* RNase P (HARP); the latter replaced the RNA-based enzyme in a small group of thermophilic bacteria but otherwise coexists with the ribonucleoprotein enzyme in a few other bacteria as well as in those archaea that also encode a HARP. Here we summarize the history of the discovery of protein-only RNase P enzymes and review the state of knowledge on structure and function of bacterial HARPs and eukaryal PRORPs, including human mitochondrial RNase P as a paradigm of multi-subunit PRORPs. We also describe the phylogenetic distribution and evolution of PRORPs, as well as possible reasons for the spread of PRORPs in the eukaryal tree and for the recruitment of two additional protein subunits to metazoan mitochondrial PRORP. We outline potential applications of PRORPs in plant biotechnology and address diseases associated with mutations in human mitochondrial RNase P genes. Finally, we consider possible causes underlying the displacement of the ancient RNA enzyme by a protein-only enzyme in a small group of bacteria.

tRNAs are transcribed as precursors with additional sequences at their 5′ and 3′ ends in all forms of life (1, 2), with a single exception (3). RNase P is the enzyme that removes tRNA 5′ extensions by endonucleolytic phosphodiester hydrolysis; 5′-end processing is a prerequisite for the further maturation of tRNAs and their utilization in cellular protein synthesis. The RNase P enzyme family is characterized by a bewildering variety of enzyme forms and represents a unique example of convergent molecular evolution (4). First discovered in bacteria, RNase P enzymes built on a structurally conserved, catalytic RNA were later found in all domains of life, and for almost 3 decades, an RNA component was considered to be a universal signature of the enzyme family (4, 5, 6). In Bacteria, the RNA only requires a small ancillary protein cofactor for enzymatic function, but in Archaea, an unrelated set of five proteins is associated with the RNA, which is expanded in eukaryal nuclear RNase P enzymes to up to 10 proteins. In early eukaryal evolution, a duplication moreover gave rise to RNase MRP, a paralog of nuclear RNase P, which is composed of a structurally related RNA associated with largely the same protein subunits. Although evidence for an “RNA-free”, “protein-only” (“proteinaceous”) form of RNase P was first reported in 1988 (7), it took 20 years until the first representative of this kind of RNase P was identified (8). Today, we know of two essentially different forms of protein-only RNase P that apparently evolved independently: one, exclusively found in Eukarya but widespread within this clade, is known by the acronym PRORP (proteinaceous/protein-only RNase P); the other one, called HARP (homolog of *Aquifex* RNase P), is exclusively found in a few bacteria and some archaea. Here, we review the discovery history and current state of knowledge of these protein-only forms of RNase P.

## The discovery of protein-only RNase P: A personal account

By the early 1980s, the pioneering work of Sidney Altman *et al.* had established bacterial RNase P as an RNA enzyme (9, 10, 11, 12), and structurally related RNA molecules were soon discovered to be associated with RNase P from yeast mitochondria (13, 14), different archaea (15, 16), and human as well as yeast nuclei (17, 18, 19, 20). Together, these findings led to the view that RNase P universally includes a catalytic RNA component, similar to its ‘big sibling’, the ribosome. In the context of this zeitgeist, the first two studies that came to conclude that the investigated form of RNase P could not have the expected RNA subunit were not driven by doubts about the existence of it. In fact, the authors initially made every effort to identify the expected universal RNA molecule in their respective model system.

In 1988, the rigorous biochemical characterization of spinach chloroplast RNase P by Peter Gegenheimer’s group led them to the heretical conclusion that this enzyme “does not contain a substantial functionally required RNA component” (7). The development of their original experimental approach, its historical and scientific context, and the general perception in the field have all been reviewed 10 years ago by Gegenheimer himself (21) and we would like to refer the reader to this review for further details. Although the work on spinach chloroplast RNase P came extremely close to the identification of the responsible protein and the overall evidence for a simple protein enzyme was compelling, not only in hindsight, the mere possibility was mostly met with strong skepticism by the field culminating in the discussion whether a protein-based RNase P, if existent at all, should then be called an RNase P (22, 23).

The story of human mitochondrial (protein-only) RNase P (mtRNase P) differs in having been overshadowed by a controversy of whether the mitochondrial enzyme is identical to the RNA-based nuclear one or not. The tRNA punctuation model of RNA processing in human mitochondria, as established in 1981, predicted two tRNA-processing endonucleases as central players in the release of all the RNA species from the polycistronic mitochondrial primary transcripts: an RNase P and a precursor tRNA (pre-tRNA) 3′ endonuclease, now called RNase Z (24, 25, 26, 27). The group of Giuseppe Attardi, leading this research, subsequently teamed up with Sidney Altman’s group to identify the responsible RNase P enzyme, and in 1985, they reported the characterization of an RNase P activity from HeLa cell mitochondria (28); a similar study on tRNA processing activities from rat liver mitochondria followed soon thereafter (29). Of note, instead of mitochondrial pre-tRNAs, both studies used *Escherichia coli* pre-tRNA^Tyr^_su3+_ as RNase P substrate, a pre-tRNA originally characterized by Altman himself in his Cambridge years (30, 31, 32) and used by his lab also in their studies of human nuclear RNase P (17, 18, 33). In the following years, the Attardi lab further characterized the assumedly mitochondrial RNase P and identified the RNA subunit of the nuclear enzyme, H1 RNA, to be associated with its activity; although apparently complete by the early 1990s (34, 35), the work was published only in 2001 (36). The concurrent discovery that RNase MRP, a presumptive primer processing endonuclease in mitochondrial DNA replication, contained a nucleus-encoded RNA (37), appeared to further support the idea of nuclear RNase P being imported into mitochondria (34); RNase MRP was later found to be a (predominantly) nuclear ribonucleoprotein (RNP) related to nuclear RNase P in structure and composition (38, 39, 40, 41, 42, 43). Yet in 1992, the lab of Witold Filipowicz found that the trace amounts of MRP RNA associated with human mitochondria were actually too minute to be consistent with its presumed mitochondrial function but rather represented a contamination derived from the large nuclear pool of the RNP (44). The resulting controversy (45) was ongoing for many years, but while a nuclear role of RNase MRP in pre-ribosomal RNA processing was increasingly substantiated (46, 47), studies on mitochondrial DNA replication never really supported its ascribed role in this process; meanwhile, RNase H1 has been identified as the enzyme responsible for primer formation (48).

In 1992, in the light of the controversial results on MRP RNA localization (44), one of the authors of this review (Walter Rossmanith), working on RNP assembly of nuclear RNase MRP at this time, was asked by his PhD supervisor (Robert Karwan) to scrutinize these findings. The experiments essentially confirmed that MRP RNA and also the additionally analyzed H1 RNA (the RNA subunit of human nuclear RNase P) were depleted to traces with increasing mitochondrial purity, while the levels of mtDNA-encoded RNA remained constant. It was decided at this point to also prepare mitochondrial extracts and compare them to nuclear enzyme preparations with regard to RNase MRP and RNase P activity. A mouse mtDNA replication origin O_H_ transcript and *E. coli* pre-tRNA^Tyr^_su3+_ were the standard substrates used (17, 18, 28, 37), but due to a concurrent collaboration with Elisabetta Sbisà and Apollonia Tullo, transcripts of the rat mtDNA replication origins (O_H_, O_L_) were included as well. However, in contrast to the nuclear enzyme preparations, mitochondrial extracts produced either no or only weak off-site cleavages when acting on the aforementioned standard substrates. Only on the rat O_L_ transcript, the mitochondrial extracts produced a strong and intriguing cleavage pattern: as this transcript included the nearby tRNA^Tyr^ and tRNA^Cys^ sequences, the observed product sizes suggested cleavages at the 5′ and 3′ ends of the two tRNAs. *In vitro* cleavage of an authentic animal mitochondrial pre-tRNA had not been reported at this time, and so the significance of the result was immediately obvious. The cleavage activities of the mitochondrial extract were swiftly reproduced on various human mitochondrial pre-tRNAs and sparked a characterization of the mtRNase P activity, still based on the assumption that it would be related to nuclear RNase P or at least contain an RNA subunit. However, the distinct substrate specificity and failure to be recognized by nuclear RNase P–specific antibodies rapidly made clear that this mtRNase P was not related to the nuclear enzyme. In the following months, inspired by the approach of the Gegenheimer laboratory (7, 49), the human mtRNase P activity was rigorously probed for the involvement of an RNA moiety. By early 1994, the experiments were largely complete and it became clear that mtRNase P did not require an RNA subunit. However, these findings were not generally welcomed by the field. For a year, the resulting manuscript was reviewed and rejected by a handful of journals one after the other, until it was finally published in this journal (50), yet only after omission of data indicating that it does not contain an RNA component and of statements on the localization of H1 and MRP RNA. The results demonstrating that mtRNase P is not an RNA-based enzyme were eventually published 3 years later (51).

Attardi’s 2001 publication (36) of H1 RNA being the subunit of mtRNase P prompted a rebuttal by W.R. (52) and re-attracted his attention to the issue. In late 2004, Johann Holzmann, a talented PhD student, started working on the purification of human mtRNase P using a substrate specific for the mitochondrial enzyme and not cleaved by nuclear RNase P. Employing a combination of partial purification of the enzyme activity and shot-gun proteomics of active fractions, a first candidate protein/gene was identified in late 2006, which was the means to identify the two remaining subunits. A reconstitution of the activity solely from recombinantly expressed proteins was achieved in late 2007 and the results published in the following year (8).

Up to three homologs of the nuclease subunit of human mtRNase P (originally called MRPP3) were right away identifiable in various eukaryal genomes (8). Those included plant and trypanosomatid homologs with targeting sequences for chloroplasts and mitochondria, consistent with prior reports on the apparently proteinaceous nature of these enzymes (7, 53, 54). Subsequent studies then showed that unlike their human homolog, the plant and trypanosomatid proteins are active as single polypeptides, and some of them even localize to the nucleus and act as an RNase P there (55, 56, 57). So human mtRNase P turned out to be an exception rather than the prototypic case of the new form of RNase P and the localization of the latter not restricted to organelles either. Consequently, the more generally applicable name PRORP was coined for the new gene family (55, 58), which finally turned out to be much more widespread than anyone had expected (59).

## Distribution and evolution of eukaryal protein-only RNase P

**Figure 1:**
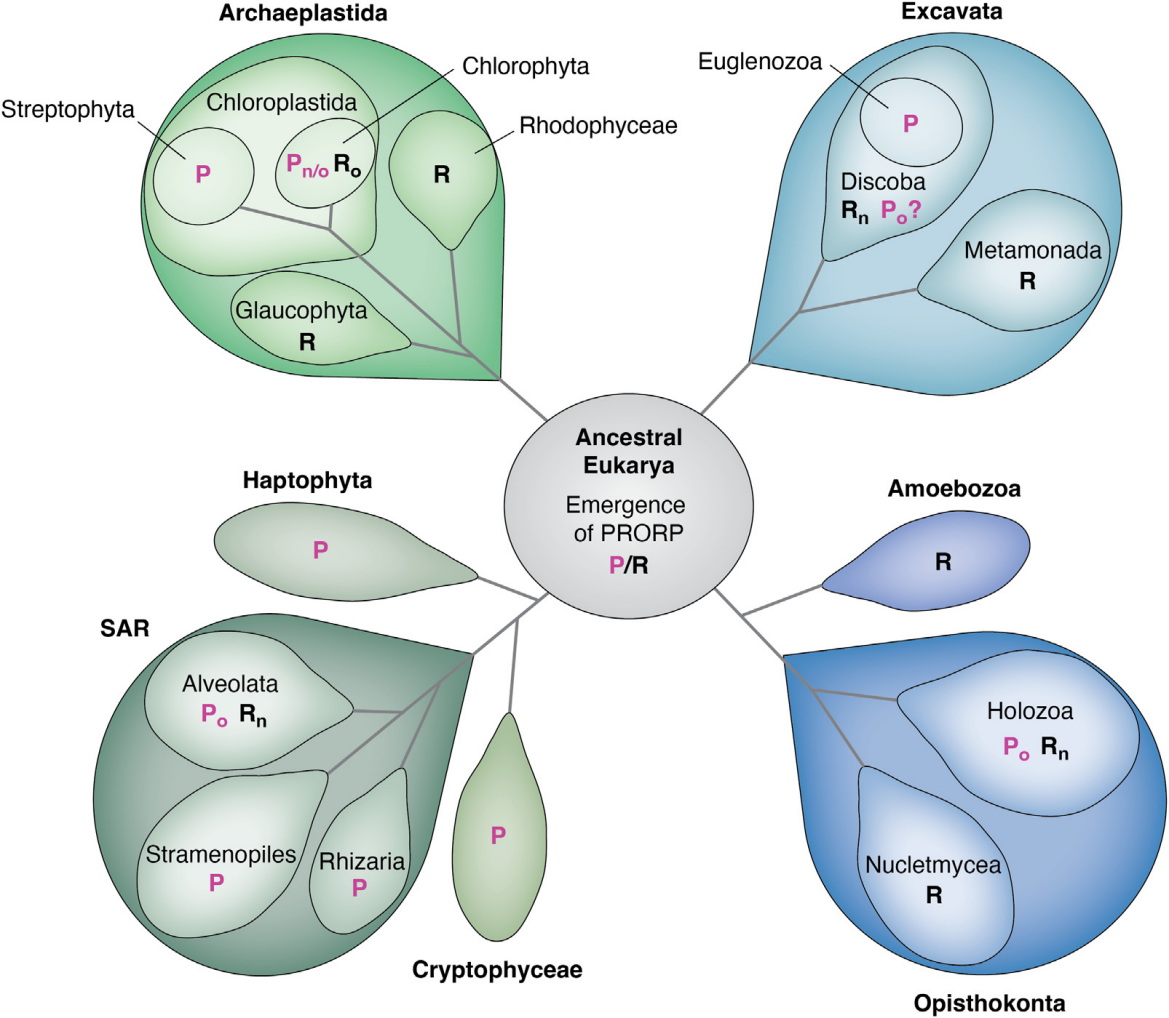
**Distribution of protein-only RNase P (PRORP) and RNA-based RNase P in Eukarya.** The occurrence of PRORP and RNA-based RNase P are indicated by P (*magenta*) and R (*black*) in the major eukaryal phylogenetic groups arranged according to their assumed relationship. Subscripts: n, nuclear; o, organellar; n/o, nuclear and organellar occurrence. The question mark (?) indicates that limited genomic data prevented definite conclusions about the occurrence of this enzyme type. PRORP, proteinaceous/protein-only RNase P.

*PRORP* genes are exclusively encoded in nuclear genomes, but the proteins can apparently be localized to the nucleus, mitochondria, chloroplasts, or other plastidial and plastid-derived organelles (59). They are present in almost all major branches of the eukaryal domain (Fig. 1); amoebozoa, fungi, and basal primary photosynthetic groups such as Glaucophyta and Rhodophyceae are the only major phyla that apparently do not have a PRORP. In land plants, stramenopiles, and trypanosomes, PRORPs appear to have replaced the RNP form of RNase P in all tRNA-processing compartments. In some instances, this change is achieved by a single PRORP being routed to all relevant compartments (nucleus, mitochondria, chloroplasts), like in the case of *Chlamydomonas reinhardtii* (60), or multiple PRORP paralogs, like in *Arabidopsis thaliana*, where one isoform localizes to both mitochondria and chloroplasts, and two are nuclear (55, 56). The latter case shows that more than one isoenzyme of PRORP can occasionally coexist within a compartment. In many Eukarya, a PRORP is nevertheless only found in either the nucleus or the organelle(s), with an RNP enzyme in the other (59). However, so far no evidence has been found for the coexistence of PRORP and RNP RNase P within the same compartment, *i.e.*, the occurrence of the two different forms of RNase P appears to be mutually exclusive in present-day Eukarya. The phylogenetic distribution of the special, multi-subunit form of protein-only RNase P found in human mitochondria (*i.e.*, a PRORP dependent on two more protein subunits) has not been investigated in detail so far. However, a mitochondrial homolog of the TRM10 methyltransferase and the modification it introduces have only been found in metazoans (61, 62), suggesting that the additional subunits were recruited within this lineage (see also below). Whether the PRORPs in all other eukaryal branches act as single-polypeptide enzymes or whether a PRORP has recruited one or more essential subunits in the other uninvestigated clades cannot be decided based on primary structure information, given that human PRORP does not display a characteristic sequence difference indicating its multi-subunit nature.

The broad distribution and a phylogenetic analysis of sequences from all major eukaryal groups suggest that PRORP evolved very early, in an organism at the root of the eukaryal tree, by fusion of an NYN domain (NYN, Nedd4-BP1/YacP nuclease) with a small cluster of tandem pentatricopeptide repeat (PPR) motifs, connected *via* a split zinc-binding domain (ZBD) (4, 59). This event seemingly took place only once in evolution, although the phylogenetic analysis also points to horizontal gene transfer events. Specifically, some groups within the SAR clade (Stramenopiles, Alveolates, and Rhizarians) apparently acquired PRORP through secondary or tertiary endosymbiosis (59). The early origin and the distribution pattern of PRORPs and RNA-based RNase P forms in the eukaryal tree also indicate that the two principally different enzyme forms have coexisted in at least those early Eukarya that gave rise to all extant species (Fig. 1). All the more surprising is the observation that this functional redundancy has apparently completely vanished, and PRORP and the RNP enzyme now appear mutually exclusive in tRNA-synthesizing cellular compartments (Fig. 1). It is unclear why one or the other form of RNase P was favored in evolution in a given organism. A possible advantage of PRORP appears to be the obviously greater evolutionary plasticity in terms of subcellular localization or horizontal gene transfer compared to a multisubunit RNP (4). While nuclear PRORPs have been adopted to function in mitochondria and plastids, there is currently no evidence that either the archaeal-type nuclear or the endosymbiont-derived bacterial-type organellar RNP RNase P have been coopted in another subcellular compartment or phylogenetic group (59).

## Eukaryal single-polypeptide protein-only RNase P

### Enzyme structure and function

Single-protein RNase P enzymes were initially identified through their similarity with the catalytic subunit of human mitochondrial RNase P (8). These enzymes are active without the requirement of any other factor both *in vitro* and *in vivo*. They were first characterized in *A. thaliana* where three PRORP paralogs occur: *At*PRORP1 localizing to mitochondria and chloroplasts and *At*PRORP2 and *At*PRORP3 both to the nucleus (55). They perform essential functions as inactivation of *At*PRORP1 or the simultaneous inactivation of *At*PRORP2 and 3 result in lethality. Functional studies demonstrated that RNP RNase P enzymes have been entirely replaced by PRORP proteins for RNase P activity in plants (56). Beyond their involvement in pre-tRNA 5′ maturation, plant PRORPs were shown to be involved in the maturation of mitochondrial mRNA ends at the level of conserved tRNA-like structure (TLS) (55), as well as in the maturation of a tRNA-snoRNA precursor in the nucleus *in vivo* (56). Likewise, in the moss *Physcomitrium patens*, three PRORP paralogs occur in nucleus, mitochondria, and chloroplasts (63). The nuclear *Pp*PRORP was shown to be non-essential; however, the data presented do not rule out the additional nuclear localization of one of the organellar *Pp*PRORPs. Similar to plants, RNase P activity is entirely attributable to two PRORP proteins localizing to the nucleus and mitochondria, respectively, in the protist *Trypanosoma brucei* (57). The ability of protein-only RNase P to functionally replace RNP RNase P was illustrated by the experimental substitution of bacterial and yeast RNP RNase P by different PRORPs *in vivo* (55, 57, 64, 65). In contrast to *Arabidopsis*, *Physcomitrium* and *Trypanosoma* encoding multiple PRORP proteins, a single *PRORP* gene is found in the unicellular green alga *C. reinhardtii*, where the RNase P RNP form is absent too. Different isoforms encoded by this single *PRORP* gene localize to all the compartments where gene expression takes place, *i.e.*, mitochondria, chloroplasts and the nucleus, thereby representing the most compact and versatile RNase P machinery described for Eukarya (60).

While occurring in a wide diversity of distantly related eukaryal lineages, PRORPs are remarkably similar. For instance, they share an average of 30% sequence similarity between *Arabidopsis* and *Trypanosoma* (59). The different PRORPs all comprise a conserved domain organization, with a tripartite architecture containing two main domains, an N-terminal PPR domain and a C-terminal NYN domain, connected by a ZBD (59, 66, 67). This molecular architecture was first revealed by crystal structures of *At*PRORP1 and *At*PRORP2 (66, 68, 69). The structures display a Λ-shaped arrangement of the three domains, where the PPR and the nuclease domains correspond to the two arms of the Λ and the ZBD forms the tip of the Λ that connects the two main domains, providing flexibility to the overall structure, as described below (70). The nuclease domain adopts a PIN-like NYN fold (PIN, PilT N-terminus; PilT, type IV pili twitching motility protein) that shows structural similarities with the nuclease domains of FLAP and DNA polymerase I nucleases (66, 67, 71, 72). In the NYN domain, the active site contains four highly conserved aspartate residues (59, 66, 68). Likewise, the ZBD is characterized by three conserved cysteines and one histidine involved in the coordination of a Zn^2+^ ion as well as by a conserved four-stranded antiparallel β sheet fold (66).

The PPR domain contains a variable number of PPR motifs (59), although all PRORPs that were structurally characterized contain five PPR motifs and one incomplete motif. PPR proteins were initially identified as a family of eukarya-specific RNA-binding proteins composed of a succession of 35 aa-long motifs of degenerate sequence, each adopting a conserved helix-turn-helix structure, which together form a superhelix (73). The identification of the canonical PPR proteins’ mode of action revealed how RNA is bound on the concave surface of the superhelix, with each PPR motif interacting with a specific nucleotide (74). Based on these findings, the PPR domain of PRORPs was proposed to be involved in pre-tRNA binding (8, 55, 66). This assumption was confirmed by a number of biochemical and biophysical approaches (69, 75, 76, 77) as well as by structure determination of the *At*PRORP1 PPR domain in complex with tRNA (78). These studies showed that the PPR domain forms specific contacts with the RNA substrate but does not follow the mode of action of canonical PPR proteins (76, 77, 78, 79, 80). Moreover, small-angle X-ray scattering studies of *At*PRORP2 in complex with pre-tRNA and *in silico* analyses suggested that PRORPs undergo conformational changes to accommodate the substrate (69). The angle of the Λ shape of PRORP in the PRORP•pre-tRNA complex opens as compared to PRORP alone. This conformational change appears to be required to accommodate the pre-tRNA substrate. In summary, structural studies of single-polypeptide PRORP enzymes revealed the conserved molecular architecture of these proteins and provided the first insights into their dynamics and RNA-binding process.

### Substrate recognition and catalytic mechanism

**Figure 2:**
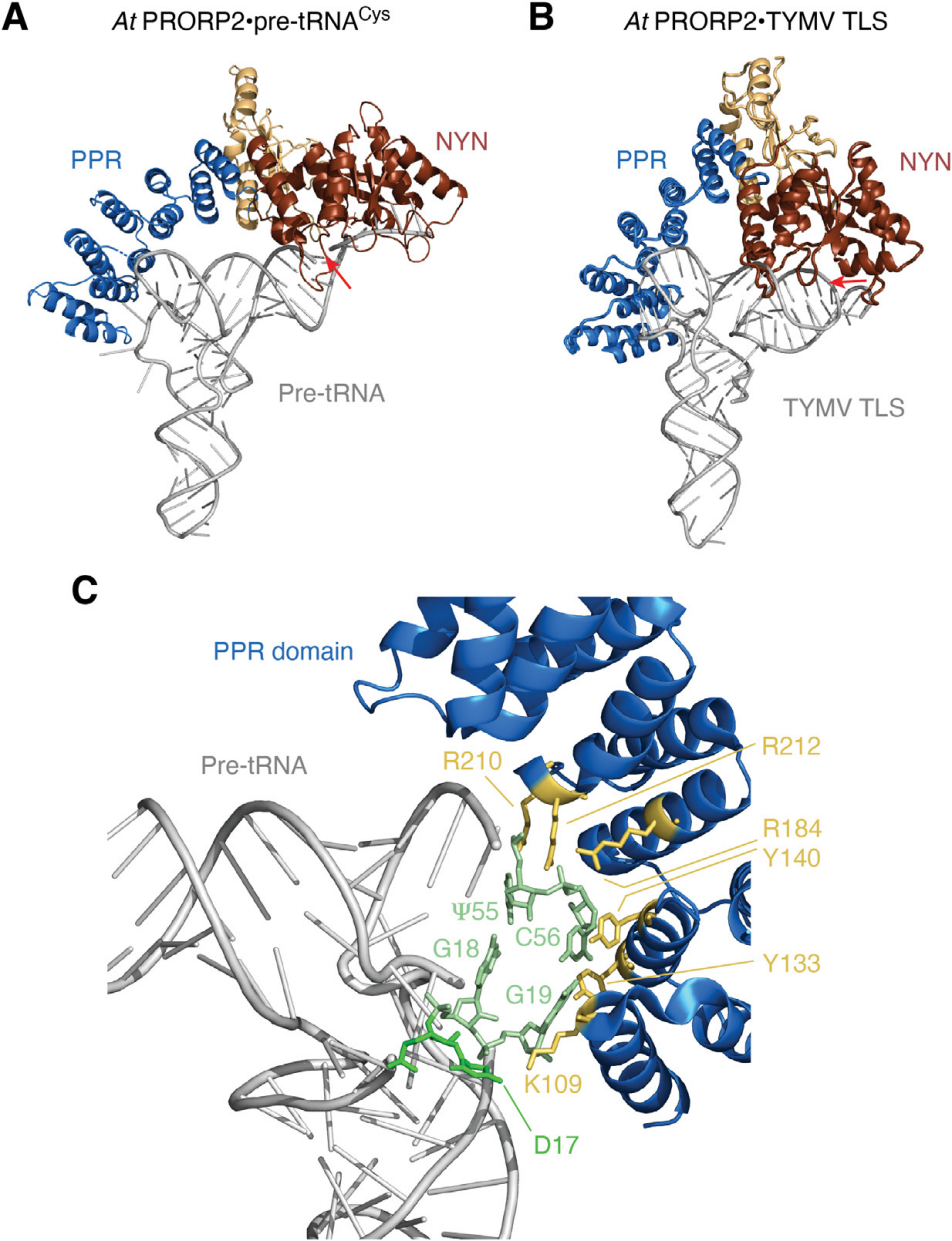
**Single-polypeptide PRORP structure and interaction with RNA substrates.***A*, model for the interaction between PRORP and a pre-tRNA^Cys^ substrate based on the crystal structure of *At*PRORP2, biochemical studies, and SAXS data of the *At*PRORP2•tRNA complex (69, 75). *B*, model for the interaction between PRORP and the tRNA-like structure of the Turnip Yellows Mosaic Virus (TYMV TLS) (93). RNase P cleavage sites, either located at the tRNA 5′ end or within the viral TLS pseudoknot structure, are indicated by *red arrows*. The PPR domain is shown in *blue*, the catalytic NYN domain in *reddish brown*, and the connecting ZBD in *beige*. *C*, details of the interaction between the PPR domain of PRORP and the tRNA ‘elbow’ were revealed by the crystal structure of the *At*PRORP1 PPR domain in complex with yeast tRNA^Phe^ (78). Specific residues of the PPR domain make direct contacts with conserved residues in the tRNA ‘elbow’. These protein and RNA residues are represented in *gold* and *green*, respectively. pre-tRNA, precursor tRNA; PPR, pentatricopeptide repeat; PRORP, proteinaceous/protein-only RNase P; SAXS, small-angle X-ray scattering; TLS, tRNA-like structure; ZBD, zinc-binding domain.

The combination of biochemical, biophysical, and structural studies has revealed that the PPR domain of single-protein PRORP enzymes specifically interacts with the ‘elbow’ of pre-tRNAs (69, 75, 78). More precisely, this interaction involves a positively charged–binding pocket that contacts the tRNA ‘elbow’ *via* its phosphate backbone and by forming base-specific interactions. Molecular details of the interaction have been provided by the structure of the PPR domain of *At*PRORP1 in complex with a pre-tRNA, revealing an interaction network involving D17 and the G18-Ψ55/G19-C56 base pairs of the pre-tRNA, and residues K109 (PPR1), Y133 (PPR2), Y140 (PPR2), and R210/184/212 (PPR3) of *At*PRORP1, respectively (Fig. 2; see also (78)). These findings are in accordance with a previous footprinting analysis that showed how some residues such as G18 or C56 make contacts with *At*PRORP1 and are essential for pre-tRNA cleavage (75). Likewise, biochemical data obtained with *At*PRORP2 and *At*PRORP3 also indicated that base-specific interactions occur between the PPR domain and the tRNA ‘elbow’ (77, 81). The structural data are in accordance with biochemical data showing the importance of specific residues in PPR motifs 2 and especially 3 for substrate binding affinity and cleavage efficiency (69, 77). Altogether, data on pre-tRNA binding obtained with different single-polypeptide PRORP proteins suggest that all these proteins share a conserved substrate-binding mode. It is remarkable to note that both PRORP proteins and RNP RNase P bind the tRNA ‘elbow’ and thus recognize their substrates in a conceptually similar manner. Indeed, structural studies of RNP RNase P showed that this enzyme type recognizes the conserved base pair G19-C56 as well (here *via* base-stacking interactions), which connects the T arm and the D arm and thereby senses the authentic architecture of the tRNA ‘elbow’ (82, 83, 84). Overall, PRORP substrate-binding studies suggest that the PPR domain has evolved to recognize and specifically bind structural features of tRNAs that are also recognized by RNP RNase P.

The most conserved feature of PRORP proteins, that actually defines the PRORP subtype of NYN nucleases, is its catalytic pocket (59, 67). Among the four highly conserved aspartates, three residues (D399, D475, and D493 in *At*PRORP1) coordinate catalytic metal ions in the vicinity of the cleavage site (66). A mechanistic model for the catalysis by protein-only RNase P has been deduced from biochemical and structural studies. Two Mg^2+^ ions that are coordinated by the conserved aspartates in turn coordinate the pro-*S*p oxygen and the 3′-oxyanion leaving group of the scissile phosphodiester in the RNA substrate (66, 85, 86, 87). While RNase P RNA was proposed to use one Mg^2+^ ion for coordinating the OH^−^ nucleophile and a water molecule from the hydration shell of a second Mg^2+^ ion to donate a proton to the 3′-oxyanion leaving group, it appears that PRORP utilizes a more conventional general acid-base mechanism that applies to classical protein metallonucleases (21, 66, 88). In conclusion, although PRORPs and RNP RNase P differ in many aspects, a clear analogy can be made between the PPR domain of PRORP and the specificity domain of the RNase P ribozyme, as well as between the NYN domain of PRORP and the catalytic domain of the RNP (21).

### Interaction partners, potential biotechnological applications

While single-polypeptide PRORP enzymes are active on their own by definition, some studies have begun to explore supramolecular interactions involving PRORP proteins. One possibility is that PRORPs may form putative tRNA maturation complexes with other tRNA processing or modification enzymes, such as RNase Z or the CCA-adding enzyme, to coordinate the biogenesis of tRNAs. Analyses in this direction were initiated for *At*PRORP1 in *Arabidopsis* mitochondria and chloroplasts (89). Immunoprecipitations using *At*PRORP1 as a bait identified proteins involved in organellar gene expression processes. In particular, a direct interaction was described between PRORP1 and mitochondrial nuclease 2 (MNU2). This interaction is mediated by a conserved PPPY motif localized between the ZBD and the NYN domain of PRORP and a tryptophan-containing (WW) protein–protein interaction domain of MNU2. Interestingly, MNU2 is a putative NYN domain nuclease that was found to be involved in the 5′ maturation of mitochondrial mRNAs (90). Although the molecular function of this PRORP1–MNU2 interaction is unknown, it is possible that PRORP1 and MNU2 cooperate in mRNA maturation, *e.g.*, for the cleavage of TLS in mRNAs or during 5′ maturation of pre-tRNAs. MNU2 could for instance trim the long leader sequences found in organelle pre-tRNAs to facilitate RNase P cleavage by PRORP1 (89). Likewise, the interaction network of nuclear *At*PRORP2/3 has begun to be explored. An immunoaffinity strategy showed that PRORP2 occurs in a complex with the tRNA methyltransferases TRM1A and TRM1B *in vivo*. This interaction appears to be mediated by pre-tRNAs. TRM1A/B enzymes are responsible for the m^2^_2_G modification at position 26 in 70% of *Arabidopsis* cytosolic tRNAs *in vivo*. A double knockout of TRM1A/B resulted in a complete rearrangement of the pool of cytosolic tRNAs. Interestingly, tRNAs receiving the m^2^_2_G modification in wild type (WT) cells were strongly downregulated in the *TRM1A/B* KO mutant and simultaneously showed impaired processing by RNase P. This finding suggests that TRM1A/B cooperate with nuclear RNase P in plants during the early steps of cytosolic tRNA biogenesis, although no component of the RNA polymerase III machinery including the La protein were identified in immunoprecipitations (91, 92). Thus, tRNA transcription and maturation do not appear to be coupled processes in plant nuclei.

An increasing number of studies has revealed the functions and mode of action of PRORPs. This fundamental knowledge begins to be used for biotechnological applications. In particular, a novel strategy aiming to generate plants resistant to viruses has been proposed (93). This approach is based on the observation that most plant viruses are RNA viruses, many of them holding a functional TLS. The removal of these structures should impede virus replication and lead to resistance against the virus. PRORP is able to cleave viral RNAs at the level of TLS *in vitro* (Fig. 2*B*). However, this antiviral process does not spontaneously occur *in vivo* because viral replication takes place in the cytosol, whereas RNase P enzymes only occur in mitochondria, chloroplasts, and the nucleus. Hence, a cytosolic variant of PRORP was generated. Its expression generates some degree of antiviral resistance *in vivo*. The evolution of resistance-breaking variants in viral populations will be slow or unlikely because of the structural constraints to maintain functional TLS in viral genomes. This approach might thus have the potential to yield durable resistance against viruses, which is of agricultural interest given the major impact of crop yield losses caused by virus infections worldwide (93).

## Eukaryal multisubunit protein-only RNase P

### The subunits of human mtRNase P

The first identified PRORP was that of human mtRNase P (8). The obvious nuclease subunit of the three-component enzyme was originally called MRPP3 (mitochondrial RNase P protein 3), as it was the third and last protein identified in the search for the remaining subunit(s) required to reconstitute the activity. It was later renamed to PRORP to reflect its evolutionary relationship with single-polypeptide PRORPs (58, 94). The other two subunits of human mtRNase P are TRMT10C (tRNA methyltransferase 10C; originally MRPP1) and SDR5C1 (short chain dehydrogenase/reductase family 5C, member 1; originally MRPP2) (8, 94). *In vitro* reconstitution of robust mtRNase P activity requires all three proteins (8, 94, 95), and mitochondrial pre-tRNAs accumulate upon depletion or knockout of any of the three (8, 96, 97, 98). Diseases associated with variants of each of the three genes (99, 100, 101) (see also below), as well as mitochondrial dysfunction and lethality upon depletion or knockout in the *Drosophila* system further confirm the essential role of all three proteins for mtRNase P function *in vivo* (102, 103). Intriguingly, the two extra subunits have additional cellular functions, beyond their role as an RNase P subunit, and so metazoan mtRNase P can be viewed more as a multi-enzyme assembly than a “simple” multi-subunit enzyme.

The role of SDR5C1 for mitochondrial RNase P is probably the least obvious. SDR5C1 is a member of the short-chain dehydrogenase/reductase (SDR) superfamily, a large group of NAD (phosphate)-dependent oxidoreductases (104). Its gene was termed *HSD17B10* to reflect the relationship to 17-β-hydroxysteroid dehydrogenases (105), but it functions as the mitochondrial short-chain l-3-hydroxy-2-methylacyl-CoA dehydrogenase that catalyzes the penultimate step in the β-oxidation of branched- and short-chain fatty acids and isoleucine (106, 107). It was also reported to be active on a wide range of alcohols and hydroxysteroids *in vitro* (108, 109, 110, 111, 112, 113); however, the physiological relevance of these findings is as unclear as that of its amyloid-β-binding properties (114, 115). SDR5C1 can be found under a plethora of names; yet because of its many unrelated roles, we advocate the use of the systematic name SDR5C1 (116), which does not refer to any specific of its diverse (presumptive) activities, associations, or functions.

**Figure 3:**
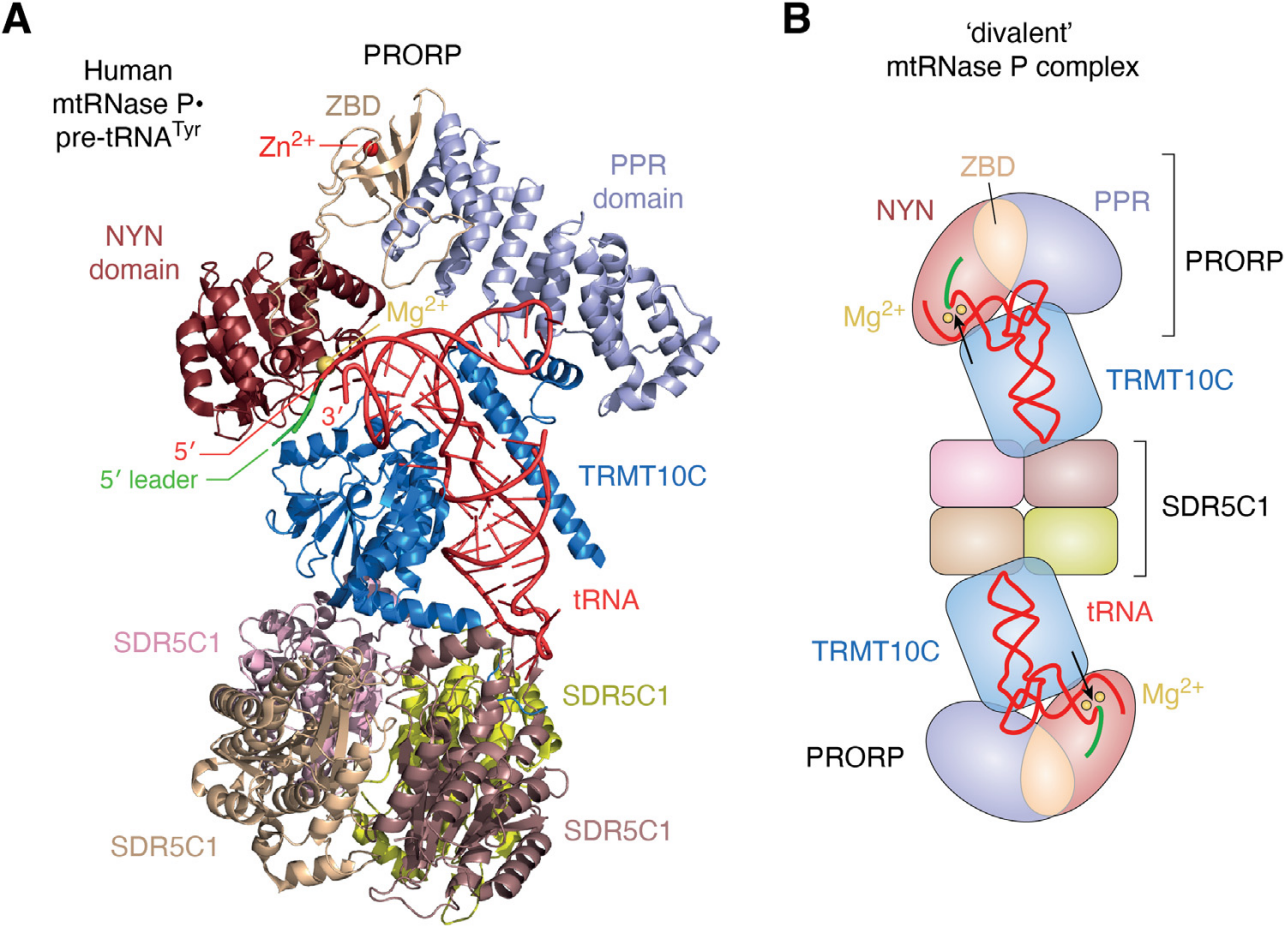
**The structure of human mtRNase P in complex with a pre-tRNA substrate.***A*, structure of human mtRNase P in complex with human mitochondrial pre-tRNA^Tyr^ as *in vitro* reconstituted from recombinant components and determined by cryo-EM (121) (PDB: 7ONU). The subunits of the holoenzyme and the domains of PRORP are individually colored. *B*, cartoon structure of a (hypothetical) ‘divalent’ mtRNase P complex (*i.e.*, a SDR5C1 tetramer with two TRMT10C molecules symmetrically attached to opposing surfaces [2:4 stoichiometry of TRMT10C:SDR5C1, as opposed to the 1:4 stoichiometry in panel *A*], each being able to concurrently bind a pre-tRNA and PRORP molecule [primarily based on biochemical evidence]), with cleavage sites indicated by *black arrows*. The color scheme is similar to that in panel *A*. pre-tRNA, precursor tRNA; PRORP, proteinaceous/protein-only RNase P.

The 27-kDa SDR5C1 polypeptide forms a homo-tetramer (117, 118) that interacts with one or two TRMT10C molecules that symmetrically attach to opposing surfaces (8, 94, 119, 120, 121) (see also Fig. 3). This stable (sub)complex is not only a subunit of mtRNase P but also represents the methyltransferase responsible for *N*^1^-methylation of purines at position 9 (R9→m^1^R9) of mitochondrial tRNAs (94). SDR5C1 itself has no detectable RNA-binding properties (94, 95), but according to the cryo-EM structure of the mtRNase P holoenzyme with bound pre-tRNA, it may form some interactions with the tip of the anticodon loop (121). The role of SDR5C1 within the methyltransferase or mtRNase P complex nevertheless is elusive and appears to be merely that of a scaffold (94, 119, 121); neither its dehydrogenase activity nor an intact NAD+/NADH-cofactor-binding site are required for the methyltransferase activity of TRMT10C or the endonucleolytic activity of PRORP.

TRMT10C is one of the three human homologs of the TRM10 group within the SpoU-TrmD (SPOUT) superfamily, a class of SAM-dependent methyltransferases defined by a deep trefoil-knotted catalytic domain (122, 123, 124). Unlike other SPOUT methyltransferases, which form dimers, TRM10 enzymes are generally monomeric (124), and TRMT10C is the so far sole exception in requiring an essential interaction partner (94). TRM10 enzymes are responsible for the G9→m^1^G9 and/or A9→m^1^A9 methylation of tRNAs in Archaea and Eukarya (124). In Archaea and the eukaryal cytosol, only some of the tRNAs carrying a G9 undergo m^1^G9 modification and m^1^A9 methylation is even more rare. However, all 19 of the 22 human mitochondrial tRNAs that have a purine at position 9 receive the m^1^A9 or m^1^G9 modification (62, 125, 126, 127).

The m^1^R9 modification has neither been found in mitochondrial tRNAs outside of the metazoan lineage nor in chloroplasts or bacteria (62). Consistently, the mitochondrial TRM10 isoform (TRMT10C) apparently originated within or at the root of the metazoan lineage, and other Eukarya apparently encode only the TRM10 isoform that modifies cytosolic tRNAs (61, 128). Also, the recruitment of SDR5C1 as a cofactor and the assumed subsequent recruitment of the TRMT10C–SDR5C1 complex as a mtRNase P subunit appear to be an early event, as the subunit composition of mtRNase P is confirmed in species as distant as humans and flies (102). For considerations on a constructive neutral acquisition of SDR5C1, we refer to our recent review (4), but we emphasize here that the obvious broadening of TRMT10C’s substrate range as a methyltransferase appears to be a consequence of its recruitment as an mtRNase P subunit (95). Thereby, it has to bind to all pre-tRNA substrates that require 5′ maturation, regardless of whether they need to be or can be methylated at all. Thereby, TRMT10C-SDR5C1 not only methylates all R9-containing mitochondrial tRNAs but also binds to and stimulates the cleavage of mitochondrial pre-tRNA^Met^ and pre-tRNA^Ser(UCN)^, the first having a C9 and the latter a noncanonical structure without the typical 2-nt connector comprising position 9 (95).

Despite its dependence on TRMT10C-SDR5C1, human PRORP does not appear to substantially differ from single-subunit PRORPs. The principal domain structure is the same and also the crystal structures of human PRORP, despite being based on N-terminally truncated fragments (129, 130), showed a Λ-shaped structure similar to *At*PRORP1 and *At*PRORP2 (66, 68, 69). However, both crystal structures displayed a substantially disordered NYN domain and lacked the catalytic metal ions (129, 130). The authors interpreted the structures as physiologically inactive conformations that would be rearranged and become cleavage-competent only upon interaction with TRMT10C-SDR5C1, although both crystalized PRORP fragments were catalytically inactive even in the presence of TRMT10C-SDR5C1. In contrast, it was recently reported that the active site of full-length PRORP is accessible to metal ions independent of its interaction partners, and PRORP is in fact also catalytically active on its own on certain pre-tRNAs, without TRMT10C-SDR5C1, though with relatively low catalytic efficiency (95) (see also below). Thus, human PRORP appears to be indeed a *bona fide* PRORP family member, not a defective pseudo-PRORP.

### Structure and mechanism of the holoenzyme; interplay of the subunits

Why does human PRORP require TRMT10C-SDR5C1, if not for the latter’s methylating activity (94), whereas other PRORPs efficiently act on their own? A structure of the mtRNase P holoenzyme in complex with a pre-tRNA substrate was expected to solve the issue. In fact, the recent cryo-EM structure of human mtRNase P was a breakthrough by representing not only the first (complete) protein-only RNase P in complex with pre-tRNA but also the first and so far, only structure of a TRM10-type methyltransferase in complex with a cognate tRNA substrate (121). The structure shows TRMT10C enclosing the long leg of the L-shaped tRNA structure by shape and charge complementarity, involving multiple interactions with all four domains of the tRNA, including specific interactions with the anticodon loop (Fig. 3*A*) (121, 131). This TRMT10C–tRNA complex sits on top of a homo-tetrameric SDR5C1 base. PRORP binds at the other end, on top of this complex, with the scissile phosphodiester bond of the pre-tRNA positioned in the active site of the NYN domain and the PPR domain interacting also *via* charge complementarity with the backbone of the T loop at the ‘elbow’ of the L-shaped tRNA (Fig. 3*A*) and not *via* recognition of specific D and T loop bases or base pairs like *At*PRORP1. However, as suggested for single-polypeptide PRORPs, human PRORP appears to adopt a more open Λ-shaped domain arrangement when bound to pre-tRNA and TRMT10C relative to its free state (121, 131). Loops of its PPR domain interact with two helices of TRMT10C’s N-terminal domain and a loop of the NYN domain with a helix of the methyltransferase domain of TRMT10C (Fig. 3*A*) (121). The published cryo-EM structure shows a ‘monovalent’ form of the holoenzyme complex (*i.e.*, able to bind and cleave one pre-tRNA molecule; Fig. 3*A*). Yet, biochemical evidence indicates a 2:4 stoichiometry of TRMT10C:SDR5C1 *in vivo* (8, 51) and in complexes reconstituted from recombinant proteins (94, 119, 120) rather than the 1:4 stoichiometry observed by cryo-EM (121). Together with cryo-EM densities from the opposite side of the SDR5C1 tetramer (121), these observations suggest that at least TRMT10C-SDR5C1 is basically able to form a mirror symmetric ‘divalent’ holoenzyme complex (Fig. 3*B*).

Although containing only a single Mg^2+^ ion, PRORP’s active-site arrangement in the mtRNase P cryo-EM structure is largely consistent with employing the same two metal ion-based catalytic mechanism described before for single-polypeptide PRORPs (121, 131). However, PRORP’s D478 side chain is tilted away from its metal-coordinating position in the cryo-EM structure when compared to the corresponding D474 side chain in the *At*PRORP1 crystal structure, which in contrast to the cryo-EM structure contains both catalytic metal ions (66, 121). This observation could indicate that the rearranged aspartates in the above-mentioned crystal structures of human PRORP fragments (129, 130) are actually the consequence of the absence of metal ions in the crystals rather than the reason for the absence of the latter. In the end, the cryo-EM structure of human mtRNase P revealed many details of the substrate-binding mechanism and an active site arrangement largely consistent with the proposed catalytic mechanism of PRORPs. Yet, the structure did not clarify how TRMT10C-SDR5C1 enables catalysis by human PRORP or why it is at all required for this purpose.

**Figure 4:**
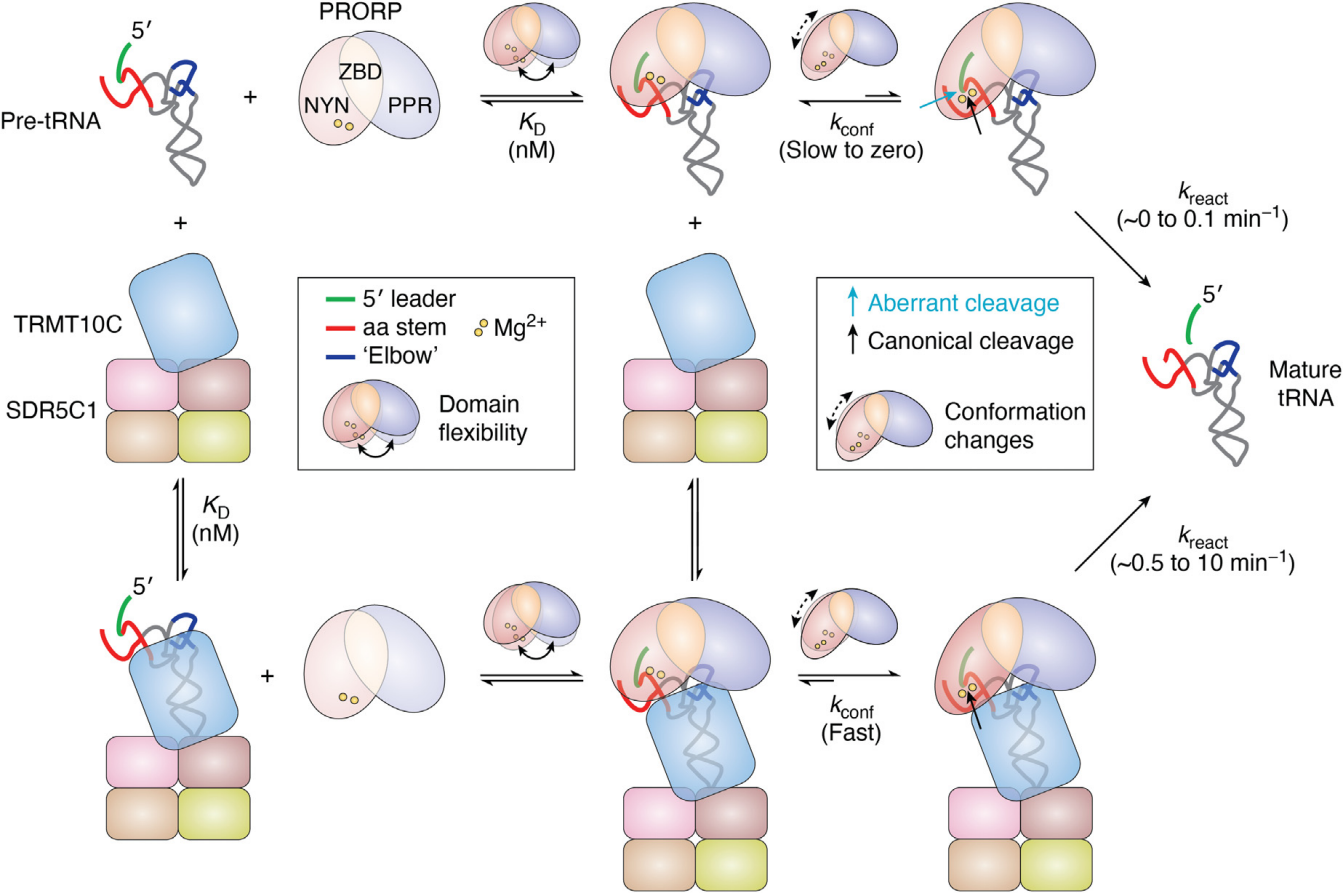
**Kinetic model of human mtRNase P; contribution of the non-nuclease subcomplex TRMT10C-SDR5C1 to tRNA 5′-end maturation by the metallonuclease subunit PRORP.** Cartoon based on a recent kinetic analysis and the available cryo-EM structure of the holoenzyme-tRNA complex (95, 121). Both, PRORP and TRMT10C bind pre-tRNA with nM affinity. The missing other component (TRMT10C-SDR5C1 or PRORP) may subsequently bind to these intermediate complexes to constitute the mtRNase P holoenzyme–pre-tRNA complex; the overall *K*_D_ of these interactions has not been determined but can be assumed to not differ substantially from the isolated pre-tRNA interactions in the subcomplexes. TRMT10C-SDR5C1 apparently mediates conformational changes in PRORP’s NYN domain that align the active site on the scissile phosphodiester bond to increase the rate and accuracy of cleavage. The 5' leader and the tRNA subdomains critically involved in the interaction with PRORP are color-coded as follows: *green*, 5′ leader; *red*, aminoacyl acceptor (aa) stem; *dark blue*, tRNA ‘elbow’ formed by the (D and) T loop. PRORP's domains are shown in differential color intensity, from light tones illustrating the least optimal orientation/conformation to darker shades indicating optimized domain conformation and orientation for productive substrate docking and efficient catalysis. In the case of the NYN domain, efficient catalysis appears to be only fully achieved upon the interaction with TRMT10C. pre-tRNA, precursor tRNA; PRORP, proteinaceous/protein-only RNase P.

Recent kinetic studies now suggest a possible mechanistic scenario for the activation of human PRORP by TRMT10C-SDR5C1 (95) (summarized in Fig. 4). As aforementioned, PRORP alone was found to not only bind pre-tRNAs with nanomolar affinity, similar to TRMT10C-SDR5C1, but to also cleave some of them with reduced efficiency. Evolutionarily, this vestigial RNase P activity of human PRORP is not too surprising, considering its obvious evolutionary descent from single-polypeptide PRORPs (59). More specifically, the findings suggest that the ancient binding mode, involving the tRNA ‘elbow’ and PRORP’s PPR domain, has basically been retained by human PRORP even outside of the holoenzyme complex. Also its metallonuclease domain is in principle correctly folded and functional, although pre-tRNA binding no longer firmly coaxes into efficient and specific catalysis, *i.e.*, some substrates are not cleaved at all, some only with lowered efficiency (and reduced precision) (95). The central role of TRMT10C-SDR5C1 is thus to increase PRORP’s cleavage rate on substrates the latter is basically able to cleave on its own, and to enable similarly efficient cleavage of mitochondrial pre-tRNAs that are not substrates for the nuclease subunit alone (95). TRMT10C-SDR5C1 apparently does so by promoting conformational changes in the nuclease domain that direct the active site to the scissile bond, changes that are slow or short-lived in the absence of TRMT10C-SDR5C1, resulting in the overall low cleavage efficiency by PRORP alone. In this manner, TRMT10C-SDR5C1 generates ≥10-fold rate enhancements, although different pre-tRNAs appear to pass through those conformational steps with varying efficiencies as reflected in the broad range of overall reaction velocities observed with different mitochondrial pre-tRNAs (95).

To put this seemingly complicated kinetic scenario in an evolutionary context, it is instructive to examine the ‘environment’ of metazoan mtRNase P. Animal mitochondrial genomes usually encode all tRNAs required for the organellar translation machinery, often in the form of the minimal set of 22. These tRNAs deviate from canonical tRNAs to different extents, some have smaller D or/and T domains, or even lack one or both of these domains, form weaker stems, and usually lack tertiary interactions that stabilize the tRNA L-shape (132, 133, 134, 135). *In vitro*, some human mitochondrial tRNAs are processed either inefficiently and/or aberrantly, or not at all, by RNP enzymes and single-subunit PRORPs including the PRORP subunit of human mtRNase P itself ((50, 95, 136) and W.R., unpublished observations). Thus, supplementation of human PRORP with an extra tRNA-binding protein complex has evidently been an important co-evolutionary event to maintain tRNA biogenesis while permitting the erosion of canonical structural features in metazoan mitochondrial tRNAs.

### Diseases associated with mtRNase P dysfunction

Rare diseases have been found to be associated with variants in each of the three genes encoding mtRNase P subunits (99, 100, 101). None of these pathogenic variants causes a complete loss of function, which in fact appears to be incompatible with life, as also indicated by the finding that the three genes are among the minimal set of the ∼2000 required for cell growth in tissue culture (137, 138). All the patients described show symptoms characteristic of mitochondrial disease, but their clinical presentations nevertheless vary with the subunit gene that is affected. The majority of patients carry mutations in *HSD17B10*, the gene encoding SDR5C1, for which also the highest number of variants is known. First classified as a metabolic disease, an inborn error of isoleucine catabolism and called MHBD deficiency (2-methyl-3-hydroxybutyryl-CoA dehydrogenase) (107, 139), mitochondrial dysfunction was later recognized to primarily underlie the observed clinical presentations and to better explain allelic variability even before the additional function in tRNA maturation had been realized (140). The primary disease manifestations are progressive neurodegeneration and cardiomyopathy, and the disease is now called HSD10 disease (101); clinical presentations vary from most fatal neonatal forms to mild late-onset forms.

Pathogenic variants in *TRMT10C* and *PRORP* were more recently identified in individuals with different clinical presentations by whole-exome sequencing. The two neonates with *TRMT10C* variants were suspected of mitochondrial disease, manifesting with lactic acidosis, deafness, hypotonia, feeding difficulties, and fatal disease progression (100). *PRORP* variants were found in four unrelated families with variable phenotypes, comprising sensorineural hearing loss, primary ovarian insufficiency, developmental delay, and brain white matter changes (99); symptoms were generally less severe than in *TRMT10C* patients.

A comprehensive comparison of the molecular consequences of the different variants is beyond the scope of this review. Moreover, due to the low number of affected individuals, with a given variant typically identified in a single individual only, the scarce data of their impact on mitochondrial tRNA processing or on mitochondrial/cellular and tissue/organ dysfunction are currently not sufficient to explain the differences in clinical presentation. Still, in all cases where this was investigated, 5′ processing of mitochondrial tRNAs was impaired, but the underlying mechanisms appear to range from a lack of expression or protein instability, problems in subunit interaction, to apparently direct impacts on the catalytic activity, or a combination of several thereof (97, 99, 100, 119, 141, 142). A mouse model with a heart and skeletal muscle–specific knockout of *Prorp*, characterized by a very short life span (98), is yet of limited value as a disease model, as a complete loss of function has not been observed in humans. This caveat also holds true for the more distant fly models (103), although here recent attempts to model human disease *via* tissue-specific knockdown rather than knockout appear more promising (143).

## Bacterial and archaeal protein-only RNase P

The discovery of PRORP in Eukarya had been a paradigm shift, demonstrating that RNase P can be a protein-only enzyme. Yet, in the 2000s, also hints for the occurrence of RNA-free RNase P in Bacteria became available (144, 145, 146). Interestingly, this type of bacterial and archaeal protein-only RNase P, finally substantiated in 2017, turned out to belong to the same superfamily of PIN domain–like nucleases as PRORP proteins (147).

### Background of bacterial protein-only RNase P (HARP) discovery

After the discovery of the RNA subunit of *E. coli* and *Bacillus subtilis* RNase P (∼400 nt, gene *rnpB*) as the enzyme’s catalytic component (12) and its small protein subunit (∼13 kDa, gene *rnpA*) as an essential cofactor *in vivo* (11), *rnpB* and *rnpA* homologs were increasingly identified in other bacteria. Initially, new *rnpB* genes were isolated by Southern hybridization techniques using heterologous *E. coli* and *B. subtilis rnpB* genes as probes (148, 149) or subsequently *via* recognizing conserved *rnpB* sequence stretches in an alignment of only seven Gram^−^ and Gram^+^ RNase P RNA sequences (149) as the basis for designing shorter hybridization probes (150). Thereafter, with the exponential growth of sequenced microbial genomes, bacterial *rnpB* and *rnpA* genes were identified bioinformatically, propelled by improving consensus structure models (*rnpB* (151, 152, 153)) and recognition of conserved sequence signatures or conserved gene synteny (*rnpA* (154, 155, 156)). Taking into account that the *rnpA* and *rnpB* genes belong to the small fraction (∼7%) of gene products that are indispensable for bacterial viability (157) and were by then indeed identified in all inspected bacterial genomes, it came as a surprise when the genome sequence of the hyperthermophilic bacterium *Aquifex aeolicus* did not uncover *rnpA* and *rnpB* genes in its small (1.55 Mbp) and densely coding genome (144, 158). In the majority of bacterial genomes, the *rnpA* gene is either cotranscribed with or in close proximity to the *rpmH* gene encoding ribosomal protein L34 (156). Even the *Hydrogenothermaceae* family members that are close relatives of *Aquifex* encode a bicistronic *rpmH-rnpA* transcription unit in their genomes (146). The gene order in *E. coli* and many other Gram^−^ bacteria is *rpmH-rnpA-yidD-yidC*, whereas this genome region is reduced to *rpmH-yidD-yidC* in the two closely related hyperthermophiles *A. aeolicus* and *A. pyrophilus* (146, 156). In a bioinformatic analysis using improved sequence- and structure-based search algorithms for *rnpB* genes, *A. aeolicus* also remained within the minor group of bacterial genomes for which no *rnpB* gene candidate could be identified (145) Yet, in contrast to the parasitic archaeon *Nanoarchaeum equitans* that compensates the lack of RNase P activity by transcribing 5′-leaderless tRNAs (3), the need for a tRNA 5′-end maturation activity was obvious in *Aquifex*: its tRNA genes are part of tRNA tandem clusters and ribosomal operons, and tRNAs with canonical mature 5′ ends could be detected in total RNA extracts from *A. aeolicus*, although RNase P activity was not detectable in *A. aeolicus* cell extracts in this early study (159). Yet, in 2008, two studies reported the detection of RNase P activity in the cell extracts of *A. aeolicus* (146, 160). Finally, the nature of RNase P in *A. aeolicus* was unraveled by biochemical purification and mass spectrometric identification of proteins copurifying with RNase P activity (147). Homologs of the protein, immediately identified in some other bacterial and many archaeal genomes, were named HARPs for homologs of *Aquifex* RNase P (147).

### HARP structure and function

**Figure 5:**
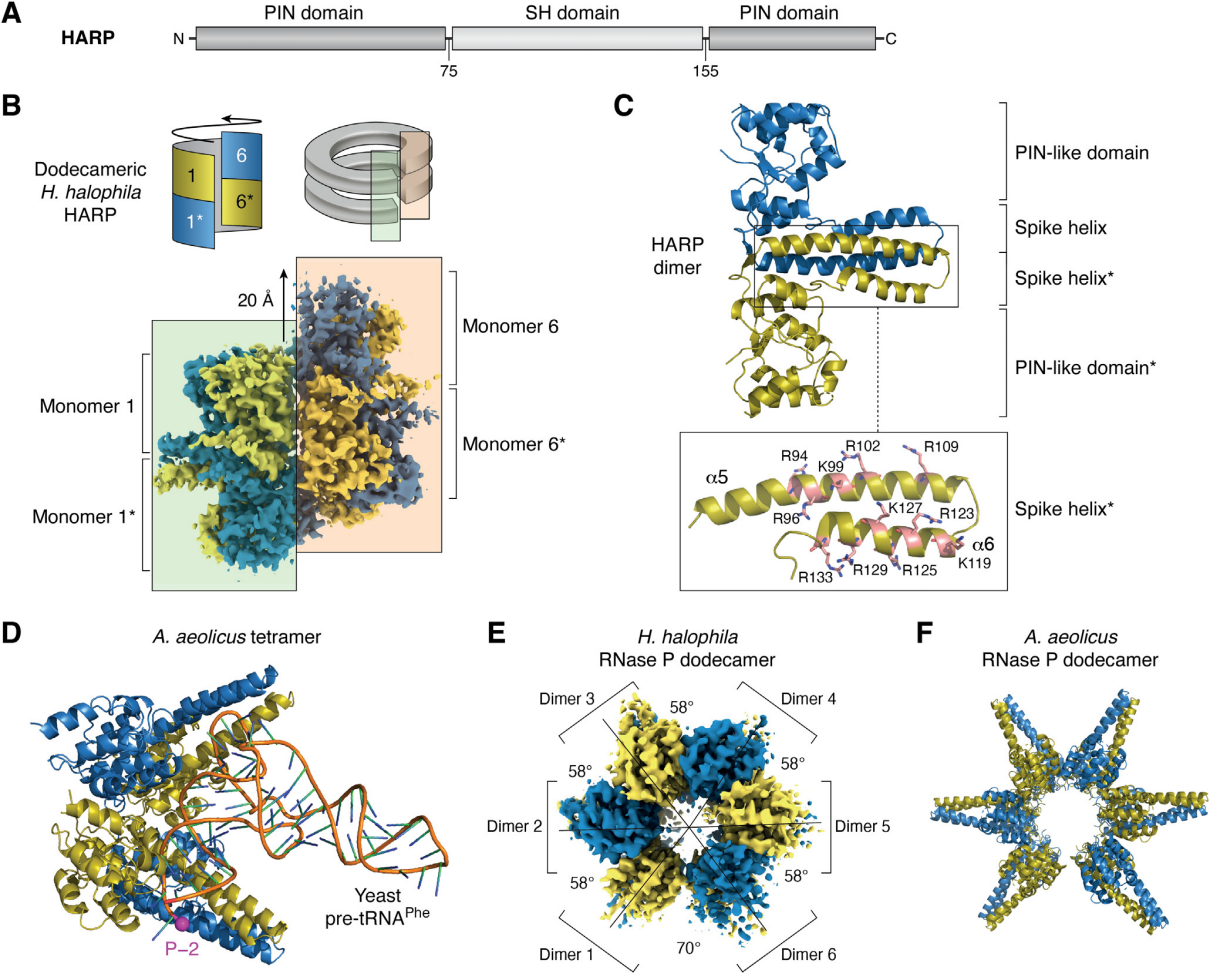
**HARP architecture and pre-tRNA binding model.***A*, domain architecture of the HARP monomer (numbering for HARP from *Halorhodospira halophila* (162)). Each monomer consists of a split PIN-like domain and an internal helical domain, termed spike helix (SH (162)) or protruding helix domain (PrH (163)). *B*, schematic presentation of the dodecameric *H. halophila* HARP architecture consisting of six dimers, where the six monomers (1–6) at the *top* and those at the *bottom* (1∗ to 6∗) resemble two split lock washers on top of each other (cartoon on the *right*). The cartoon on the *left* and the cryo-EM structure below illustrate the interface between dimers 1 and 6 (monomers shown in *olive* and *blue*). In contrast to interfaces between internal dimers (1/2, 2/3, 3/4, 4/5, and 5/6), dimer 6 is vertically shifted by ∼20 Å relative to dimer 1, which prevents tRNA binding at the interface of dimers 1 and 6; the corresponding dimer 1/6 interfaces in the cryo-EM structure and the double split lock washers cartoon above are correlated *via* the transparent *light green* and *apricot*-colored rectangles. The steric clash between dimers 1 and 6 also explains why oligomerization is restricted to dodecamers. *C*, structure of a HARP dimer (*A. aeolicus* RNase P (163)). The spike helices α5 and α6 of two HARP monomers assemble into a four-helix bundle and such dimers represent the basic functional module of HARP enzymes. The SH domain of the *olive* monomer is shown enlarged at the *bottom*, illustrating its decoration with positively charged amino acid side chains (numbering for *A. aeolicus* RNase P). *D*, model of an *A. aeolicus* tetramer with a yeast pre-tRNA^Phe^ molecule (2-nt 5′-leader) bound (163) (PDB file kindly provided by Takamasa Teramoto). The phosphate at nt-2 is marked by the *magenta sphere* and labeled P-2. The picture accentuates the close proximity of the T loop to the SH domains of the *upper* dimer. The pre-tRNA would be processed by the metallonuclease domain of the olive monomer of the *bottom* dimer. *E*, top view on the cryo-EM structure of the *H*. *halophila* HARP dodecamer; angles between individual dimers are indicated. The SH domain structure was not fully resolved due to low electron density (see also structure in panel (*B*)), suggesting conformational flexibility. *F*, top view on the cryo-EM structure of the *A*. *aeolicus* RNase P dodecamer with fully resolved SH domains (163). Monomers of each dimer are colored in *blue* and *olive* in the molecular structures presented in panels (*B*–*F*). HARP, homolog of *Aquifex* RNase P; pre-tRNA, precursor tRNA.

RNase P activity is associated with a single polypeptide of ∼23 kDa in *A. aeolicus* and other members of the family *Aquificaceae* within the phylum Aquificae; *Aquificaceae* are Gram^−^, thermophilic or hyperthermophilic, motile and non-sporulating bacteria, which are part of the microbiome of terrestrial and marine hot springs worldwide (161). The ∼23 kDa protein-only RNase P enzymes (HARPs) are members of the superfamily of PIN domain–like metallonucleases, within which they have been classed with the PIN_5 cluster (VapC-like structural group [VapC, virulence-associated proteins C] (72)). Each HARP monomer is a metallonuclease domain with an inserted, small helical domain (Fig. 5*A*), termed spike-helix (162) or protruding helix (163). HARPs form homo-oligomers, the largest being dodecamers. HARP dodecamer ‘rings’ consist of two layers of six monomers each, where monomers sitting on top of each other (colored in olive and blue) form dimers (Fig. 5, *B*–*D*). This architecture, illustrated for the cryo-EM structure of a HARP dodecamer of the bacterium *Halorhodospira halophila* (Fig. 5*B*, bottom) (162), resembles a distorted double donut or two split lock washers slightly displaced on top of each other (Fig. 5*B*, upper right cartoon). As the ‘helical pitch’ of the dodecamer is rather small, the first and sixth dimer collide and thus prevent any expansion of the dodecameric assembly (Fig. 5*B*, upper left cartoon). The spike helices of two HARP monomers assemble into a four-helix bundle and such dimers represent the basic functional module of HARP enzymes (Fig. 5*C*). The dodecamer, which is also the major oligomeric form in the case of the HARPs studied so far (162, 163, 164), can thus also be described as an assembly of six dimers. Structural models (162, 163), supported by an X-ray structure of a HARP dimer in complex with a pre-tRNA substrate (164), suggest that one dimer contacts the tRNA ‘elbow’ region and an adjacent HARP dimer docks the scissile phosphodiester bond at the 5′-end of the acceptor stem to one of its metallonuclease active sites (Fig. 5*D*). This model predicts that tetramers consisting of two dimers are the minimal catalytically competent entity of HARPs. Neighboring dimers interact side-by-side through mainly polar contacts with 900 Å^2^ of surface burial (162, 163, 164).

The dodecamer structures of *H. halophila* (Fig. 5*E*) and *A. aeolicus* (Fig. 5*F*) also revealed a gap and a larger angle between the first and sixth dimer, such that these two dimers are predicted to be incapable of forming a productive (pre-)tRNA-binding site. As a consequence, the dodecamer could at most bind ten pre-tRNAs simultaneously (162, 163). Considering that the two tRNAs potentially binding to each tetramer would come quite close to each other in the model by Teramoto *et al.* (163), it remains to be seen how many pre-tRNAs the dodecamer is able to accommodate simultaneously. The conformation of a HARP dimer in complex with a pre-tRNA (164) differed from that of HARPs in the absence of (pre-)tRNA (162, 163, 164). This observation and the lower electron density of *H. halophila* HARP in the spike helix domains (Fig. 5, *B* and *E*) suggests that the spike helix domain of HARPs has increased flexibility and may undergo conformational changes upon substrate binding. At present, it is also unclear if HARP tetra-, hexa-, octa-, or decamers, which are present in HARP preparations as well (147, 165), display activities comparable to those of the corresponding dodecamers.

Binding of pre-tRNA to HARPs appears to be dominated by electrostatic contacts, involving a series of Arg and fewer Lys residues (Fig. 5*C*, lower part) (162, 163, 164)). This pattern is not unusual for proteins interacting with the tRNA ‘elbow,’ while RNA-based enzymes (bacterial RNase P, T-box riboswitches) rely primarily on RNA base stacking interactions (ref. (163) and Fig. S12 therein). Positively charged side chains are clustered in the spike-helix domain of HARPs and it is unclear at present whether some of these key Arg/Lys residues of HARPs are involved in tRNA T loop binding at one dimer as well as in acceptor stem/5′-leader binding at the partner dimer. So far, molecular details on how HARPs recognize the acceptor stem and 5′ leader are unavailable.

### Phylogenetic occurrence of HARPs

HARPs sporadically occur in five other of the 36 bacterial phyla beyond Aquificae. Homologs were identified in some Proteobacteria, Thermodesulfobacteria, Nitrospirae, Verrucomicrobia, Planctomycetes, and a few unclassified bacteria (147, 166). Most of these bacteria also encode *rnpA* and *rnpB* genes and we recently reported that both types of RNase P, RNA-based and HARP, are potentially able to contribute to the essential tRNA 5′-end maturation activity in the thermophilic bacterium *Thermodesulfatator indicus* (165). A more wide-spread, yet patchy occurrence of *harp* genes was observed in Archaea. Many homologs were identified among Euryarchaeota. All HARP-encoding archaeal genomes additionally code for the RNA and protein components of the canonical archaeal RNase P (147, 166). To study the role of HARPs in archaeal tRNA 5′-end maturation, *harp* gene knockouts were constructed in the two Euryarchaeota *Haloferax volcanii* and *Methanosarcina mazei*. These mutant strains showed WT-like growth behavior under standard conditions as well as temperature and salt stress (*H. volcanii*) or nitrogen deficiency (*M. mazei*) (167). In contrast, deletion of the RNase P RNA gene in the polyploid *H. volcanii* failed and depletion of RNase P RNA to ∼20% of the WT level caused tRNA processing and severe growth defects (168). Taken together, these observations argue against a housekeeping RNase P function of HARPs in Archaea but rather point to specialized, yet unknown functions in archaeal (t)RNA metabolism (167).

RNase P processing activity was investigated for several archaeal HARPs and HARPs from bacteria co-expressing an RNA-based RNase P (147, 165, 167), either by analyzing recombinant HARPs for pre-tRNA processing activity *in vitro* or by HARP growth rescue of a conditionally lethal *E. coli* strain with repressible expression of its endogenous RNase P. HARP activities varied, usually being lower than that of *Aquifex* RNase P and some of the tested bacterial HARPs failed to show activity in one or both of the two assays (165). Yet, this failure may not necessarily indicate a fundamental loss of catalytic capacity but may be attributable to defective folding of the recombinant HARP *in vitro* or in the heterologous *E. coli* host. Remarkably, all three tested archaeal HARPs (147, 167) and three of the analyzed bacterial HARPs (165), as well as another bacterial HARP analyzed elsewhere (164), displayed at least some RNase P activity either in *in vitro* pre-tRNA processing assays or by genetic complementation of the *E. coli* RNase P depletion strain. These observations suggest that HARPs are able to recognize pre-tRNAs and to catalyze the RNase P–specific hydrolysis reaction, even if the RNA-based RNase P variants play the housekeeping role in the respective organisms.

The function of HARPs in Archaea is unclear. About one-fifth of bacterial and archaeal proteins in the PIN domain–like superfamily represent components of toxin-antitoxin systems (67, 166, 167). The VapC-like group, to which HARPs have been assigned (72), includes bacterial and archaeal toxins that cleave mRNAs, rRNAs, or tRNAs to inhibit translation (reviewed in (67, 167, 169)). As such an RNA-degradative function is unlikely for HARPs, other possibilities have to be considered, for example, a role as back-up RNase P activity under conditions that negatively affect biogenesis or activity of the RNA-based housekeeping RNase P or a (pre-)tRNA storage function that may permit cells to quickly resume protein synthesis after a translational shutdown. Another homo-oligomeric tRNA-binding protein is the dodecameric L-seryl-tRNA^Sec^ selenium transferase (SelA). SelA is a pyridoxal phosphate–dependent enzyme that converts a UGA-decoding tRNA^Sec^ (SelC) charged with serine and selenophosphate to selenocysteinyl (sec)-tRNA in bacteria (170). This enzyme forms a ring-shaped structure (170) and can bind up to ten tRNA^Sec^ molecules; recognition and catalysis of each bound tRNA requires the involvement of four SelA subunits (170). This enzyme form found in many bacteria is a further example beyond HARPs to illustrate the principle how protein monomers can generate complex functionalities through assembly into large homo-oligomeric architectures. SelA homologs of bacteria that have lost the Sec-decoding trait (*e.g.*, *Helicobacter pylori*) encode SelA homologs lacking the N-terminal tRNA^Sec^-binding domain, pointing to another function of SelA in those bacteria (171).

### Why replace RNP RNase P in *Aquificaceae*?

As outlined above, HARPs are wide-spread among archaea and basically capable of catalyzing the RNase P reaction, but the RNP enzyme carries out the housekeeping RNase P function in Archaea. In this context, it seems a plausible evolutionary scenario that the ancestor of the *Aquificaceae* acquired a *harp* gene *via* horizontal gene transfer from an archaeon and enhanced the enzyme's efficiency by a limited number of mutations. The tRNA 5′-maturation efficiency of HARPs in thermophilic *Aquificaceae* may have sufficed in view of slower generation times but could well be insufficient for tRNA maturation in fast-growing intestinal bacteria such as *E. coli*. The loss of the *rnpA* and *rnpB* genes in the ancestor of the *Aquificaceae* may have simply been a chance event without severe evolutionary constraints to replace the RNP enzyme. Yet, if driven by potential evolutionary constraints, then the strongly condensed *Aquificaceae* genomes (1.6. Mbp for *A. aeolicus*) might be invoked. However, as recently argued (4), the genome space saved upon replacing the *rnpA*/*rnpB* genes with a *harp* gene is quite negligible, and deleting one of the two 16S-23S-5S rRNA operons of *A. aeolicus* would have saved considerably more space. Another line of thought concerns the high growth temperature of *Aquificaceae* (up to 95 °C for *A. aeolicus*), at which folding of RNase P RNA, coordination of catalytic metal ions, and RNP assembly might pose challenges. However, other hyperthermophilic bacteria such as *Thermotoga maritima*, growing at temperatures of up to 90 °C (172, 173) and maintaining motility at up to 105 °C (174), express a canonical bacterial RNP enzyme. Moreover, apart from ribosomes, *A. aeolicus* expresses other noncoding RNAs, including the signal recognition particle RNA, tmRNA, and 6S RNA, which evidently adopt their characteristic folds and form functional RNPs with their respective protein interaction partners at high temperatures. A possible constraint might have had its origin in the coordinate expression of RNase P RNA and RnpA protein as well as holoenzyme assembly. These processes might be more complicated and possibly more prone to failure at very high temperature than homo-oligomerization of HARPs.

## Protein-only RNase P: A paradigm of convergent evolution

One of the most remarkable aspects about the RNase P enzyme family is the use of different macromolecular classes as principal catalyst, RNA in the ancient RNP enzymes, and protein in the two more recently evolved protein-only forms. Notably, all forms of RNase P nevertheless employ two-metal ion mechanisms for phosphodiester hydrolysis, the protein-only RNase P enzymes utilizing carboxyl groups of aspartate side chains, the RNA-based enzymes phosphate and uracil oxygens as ligands in the coordination sphere of the two catalytic Mg^2+^ ions (66, 82, 83, 84, 121, 162, 175); and despite their independent origin, the different protein-only enzyme types employ the same principal nuclease fold for this purpose. Similar strategies are also employed to solve the problem of specifically recognizing an entire family of cellular macromolecules (tRNAs) and directing cleavage to the correct site. All forms of RNase P apparently “sense” the conserved distance between the structurally conserved tRNA elbow and the acceptor stem 5′-end (77, 82, 83, 84, 121, 175, 176, 177), even though employing different physicochemical strategies. While base-stacking interactions dominate the interaction of the catalytic RNA with the tRNA elbow (82, 83, 84, 175), electrostatic interactions with positively charged amino acid side chains prevail in the binding of the protein enzymes (78, 121, 162, 163, 164), although a tyrosine stacking contact is involved in tRNA elbow recognition by PRORPs too (78, 121). In the end, it remains one of the key questions in the field, why tRNA 5′-end processing represents such a unique playground for convergent evolution, where novel answers to a simple problem continued to evolve despite the presence of a proven solution.

## Conflict of interest

The authors declare that they have no conflicts of interest with the contents of this article.
